# Imaging the neurohiv brain in animal models: past, present, and future—toward longitudinal, whole-brain aging phenotypes

**DOI:** 10.3389/fnagi.2026.1767149

**Published:** 2026-07-01

**Authors:** Paddy Ssentongo, Anna Ssentongo

**Affiliations:** 1 Division of Infectious Diseases, Department of Medicine, Penn State Health Milton S. Hershey Medical Center, Hershey, PA, United States; 2 Division of Epidemiology, Department of Public Health Sciences, Penn State College of Medicine, Hershey, PA, United States

**Keywords:** brain aging, diffusion MRI, EcoHIV, multimodal imaging, neuroHIV, neuroimaging

## Abstract

Animal models are essential for defining mechanisms of HIV-associated brain injury in the antiretroviral therapy era, where neurocognitive impairment and brain aging have replaced opportunistic infections as dominant clinical concerns. Humanized mice, EcoHIV murine systems, and SIV or SHIV nonhuman primates now enable increasingly sophisticated brain imaging across *in vivo* and *ex vivo* scales. Structural and diffusion MRI, perfusion imaging, manganese-enhanced MRI, and multiphoton and endoscopic optical methods provide complementary sensitivity to tissue integrity, hemodynamics, cellular dynamics, and neurovascular coupling. *Ex vivo* clearing and light-sheet microscopy further support whole-brain mapping and atlas-based integration with histology. We synthesize how neuroHIV imaging has progressed from structural surrogates to quantitative, multimodal pipelines and highlight a central gap for neurocognitive aging research. Longitudinal diffusion and perfusion MRI in SIV-infected macaques demonstrate that within-animal imaging can capture infection-associated trajectories. In contrast, systematic *in vivo* whole-brain neuroimaging remains limited in widely used murine neuroHIV models, particularly EcoHIV, despite strong behavioral and virologic phenotyping. We argue that the next phase of the field requires standardized, longitudinal, multiscale pipelines that couple high-field MRI and targeted molecular imaging with mesoscopic optical and endoscopic approaches and whole-brain three-dimensional mapping frameworks. Such integration should enable imaging-derived brain aging phenotypes in neuroHIV models, clarify mechanisms linking persistent inflammation to neurovascular and circuit dysfunction, and accelerate intervention-ready biomarkers aligned with aging cohorts of people with HIV.

## Introduction

Despite effective systemic viral suppression, HIV persists in the central nervous system and is linked to neurocognitive impairment, mood disorders, and clinically meaningful changes in brain structure and function (Alford and Vera, 2018). Direct mechanistic work in humans is constrained by ethical limits on invasive sampling, heterogeneity in antiretroviral therapy and comorbidities, and the long timescale of aging (Lagathu et al., 2017; Rodes et al., 2022). Animal models that recreate key aspects of HIV neuropathogenesis are therefore indispensable for dissecting causal pathways, testing interventions, and validating noninvasive biomarkers of brain health (Waight et al., 2022).

NeuroHIV models span several species and virologic strategies. EcoHIV introduces a chimeric virus that productively infects conventional mice and induces learning and memory deficits (Gu et al., 2018; Potash et al., 2005). Humanized mice engrafted with human immune cells or tissues support HIV-1 infection and can model aspects of central nervous system involvement (Honeycutt et al., 2015). Simian immunodeficiency virus and SHIV infection of macaques provides the closest approximation of human neuroHIV, including complex brain anatomy and the capacity for longitudinal studies relevant to aging trajectories on chronic ART (Estes et al., 2018; Hsu et al., 2018).

This mini review summarizes how brain imaging in animal models of HIV has evolved from early structural and neuropathology-correlated surrogates to increasingly quantitative, multimodal pipelines that can be deployed longitudinally (Li et al., 2011; Zhang and Li, 2013). We highlight the past and present using primary *in vivo* neuroimaging examples in mechanistic models of neuroHIV, specifically diffusion and perfusion MRI, and then focus on the future need for repeated, within-animal imaging across months to years to disentangle infection effects from normal aging trajectories and to support intervention-ready biomarkers of neurocognitive risk. In people with HIV receiving antiretroviral therapy, neuroimaging studies consistently demonstrate abnormalities in white matter microstructure, cortical and subcortical atrophy, and altered cerebral perfusion, often associated with cognitive impairment and accelerated brain aging. These imaging signatures provide a critical translational framework, as diffusion MRI, volumetric MRI, and perfusion measures in animal models can be directly aligned with these human-derived phenotypes.

## Past and present: what has been demonstrated with *in vivo* imaging

### Nonhuman primates: longitudinal MRI as proof of concept for trajectory-level neuroHIV phenotypes

The strongest primary evidence for longitudinal *in vivo* brain imaging in neuroHIV-relevant systems comes from SIV-infected macaques, where MRI can be repeated across infection stages to quantify within-animal change. Prior studies have demonstrated region-specific white matter microstructural injury (reduced fractional anisotropy), alterations in cerebral blood flow, and subcortical volume changes that parallel cognitive decline in SIV infection, although most studies remain limited by small sample sizes (often n < 10), heterogeneous infection paradigms, and inconsistent use of chronic antiretroviral therapy (Li et al., 2011; Zhang and Li, 2013). In a preliminary longitudinal study, diffusion tensor imaging and perfusion MRI track infection-associated alterations in white matter microstructure and cerebral blood flow, providing a template for trajectory-based outcomes rather than cross-sectional comparisons (Li et al., 2011). Subsequent work reinforced the feasibility of quantitative MRI approaches in SIV macaque brains and the utility of diffusion-derived measures as imaging signatures of brain injury in this model (Zhang and Li, 2013). Together, these studies establish that neuroHIV-relevant infection paradigms can support repeated measures imaging, which is an essential prerequisite for neurocognitive aging inference.

What remains limited is that, compared with human neuroHIV imaging, the nonhuman primate literature remains relatively small in sample size and heterogeneous in acquisition and processing. Longitudinal imaging under chronic antiretroviral therapy and aging-relevant timescales remains an important frontier.

## Murine systems: feasibility is high, but systematic neuroHIV imaging remains uneven

Murine systems offer unmatched scalability for aging studies and mechanistic experiments (Flurkey et al., 2007). However, the depth and consistency of published in vivo neuroimaging vary substantially across murine neuroHIV models. Manganese-enhanced MRI has been used to detect region-specific neuropathology in chronically virus-infected humanized mice at high spatial resolution, providing direct evidence that quantitative in vivo neuroimaging can capture HIV-associated brain injury signals in small animals (Boska et al., 2014; Bade et al., 2016). These studies establish a clear proof of principle that neuroHIV-relevant murine infection contexts are compatible with advanced brain imaging.

In addition, limited studies have explored structural MRI and diffusion-based approaches in other murine neuroHIV contexts, although findings have been heterogeneous and often lack replication. Few studies have systematically evaluated imaging–behavior relationships, and negative or inconclusive results are rarely reported, limiting assessment of reproducibility and effect size across models.

EcoHIV mice, by contrast, are extensively characterized at the behavioral, virologic, and immunologic levels (Xie et al., 2024; Kim et al., 2024; Dong et al., 2020), yet systematic published in vivo whole-brain neuroimaging datasets, including longitudinal MRI, diffusion imaging, functional MRI, or PET, remain sparse or absent as a reproducible body of work (Bertrand et al., 2019). This discrepancy represents a key translational gap, as EcoHIV is scalable, biologically relevant, and compatible with longitudinal imaging and intervention studies, yet remains underutilized.

EcoHIV infection is associated with microglial activation, synaptic alterations, and neuroinflammatory signaling, as demonstrated in prior experimental studies, alongside measurable deficits in learning, memory, and affective behavior (Kim et al., 2024). These phenotypes suggest plausible imaging correlates, including hippocampal volume loss, reduced white matter integrity, and altered perfusion or neurovascular coupling. Despite this strong biological and behavioral foundation, a targeted literature search (e.g., PubMed using “EcoHIV AND MRI OR diffusion OR PET”) reveals no reproducible body of longitudinal whole-brain neuroimaging studies in this model, highlighting a clear gap between mechanistic characterization and imaging implementation.

Taken together, nonhuman primate studies establish the longitudinal imaging paradigm, while murine studies demonstrate technical feasibility. EcoHIV, in turn, represents a scalable and biologically relevant model in which advanced imaging approaches remain underutilized.

### Imaging modalities: precision, tradeoffs, and aging relevance

Rather than catalog every approach, we emphasize modalities most directly aligned with neurocognitive aging. These are approaches that can be repeated longitudinally, quantify trajectories, and link brain changes to behavior and molecular aging.

### Structural MRI and diffusion imaging

High-field structural MRI provides whole-brain coverage and enables quantification of regional volumes and ventricular changes. Diffusion tensor imaging adds sensitivity to microstructural white matter injury, which is highly relevant to neuroHIV and aging phenotypes. In SIV macaques, diffusion and perfusion MRI have already been applied longitudinally, supporting trajectory-based modeling. From an aging perspective, diffusion and structural trajectories can be modeled as slope phenotypes and later paired with imaging-derived brain age frameworks (Li et al., 2011; Zhang and Li, 2013).

Importantly, diffusion and structural MRI metrics in neuroHIV studies have been linked to behavioral performance in select studies, where reductions in white matter integrity correlate with impairments in working memory and executive function, supporting a direct brain–behavior relationship, consistent with observations in human neuroHIV cohorts (Khobo et al., 2025). These relationships remain underexplored in murine systems but represent a critical opportunity for longitudinal designs, where imaging trajectories can be directly mapped onto cognitive decline over time. These measures could further be integrated with emerging brain-age modeling frameworks to quantify accelerated neurocognitive aging in neuroHIV.

### Perfusion and hemodynamics as an aging-relevant axis

Perfusion MRI adds a vascular dimension relevant to both aging and HIV, including hemodynamic disruption (Li et al., 2011). A major conceptual challenge for translation, particularly for functional MRI, is that BOLD signals depend on intact neurovascular coupling, which is influenced by arousal, motion, and anesthesia. At a mechanistic level, HIV-associated vascular dysfunction may reflect endothelial injury, blood–brain barrier disruption, pericyte loss, and chronic perivascular inflammation, all of which can impair neurovascular coupling. These processes may manifest in imaging as reduced perfusion, altered hemodynamic responses, and regional hypoxia, providing a direct link between molecular pathology and imaging-derived phenotypes.

### Manganese-enhanced MRI

Manganese-enhanced MRI provides high-resolution functional contrast in small animal brains and has been used to reflect HIV-associated neuropathology in murine systems (Bade et al., 2016; Bade, 2015). Toxicity and dosing constraints remain important limitations, particularly for longitudinal aging designs.

## Mesoscopic optical and endoscopic imaging, and whole-brain vascular mapping

Mesoscopic optical imaging approaches provide cellular- and microvascular-scale access to brain structure and function, enabling direct interrogation of neuroimmune interactions and neurovascular coupling that plausibly link chronic inflammation to accelerated brain aging (Xu et al., 2024; Prevedel et al., 2025). Conventional two-photon microscopy has enabled longitudinal cellular imaging *in vivo* but is largely restricted to superficial cortical layers, limiting access to deeper cortical and subcortical structures that are central to cognition and affect (Prevedel et al., 2025). In contrast, three-photon microscopy, by using longer excitation wavelengths and higher-order nonlinear excitation, overcomes many of these depth limitations and enables minimally invasive, high-resolution imaging of deep cortical and subcortical regions in the intact mouse brain (Prevedel et al., 2025).

Endoscopic optical imaging provides a complementary strategy for deep-brain access by allowing targeted visualization of subcortical nuclei, hippocampus, and basal forebrain through minimally invasive probes (Bocarsly et al., 2015; Turtaev et al., 2018). Although spatial coverage is restricted relative to wide-field optical methods, endoscopic approaches enable repeated, cell-resolved measurements from anatomically defined regions that are otherwise inaccessible, making them attractive for hypothesis-driven studies of circuit dysfunction and neuroinflammation in murine disease models (Malvaut et al., 2020).

Beyond optical methods, transcranial three-dimensional ultrasound localization microscopy (ULM) has emerged as a powerful, noninvasive approach for whole-brain microvascular imaging in mice. ULM enables mapping of the cerebral microvasculature and blood flow through the intact skull at microscopic spatial resolution and high temporal resolution, with quantitative measures of vessel density and flow velocity registered to standardized brain atlases (Demeulenaere et al., 2022). This capability fills a critical gap between cellular optical imaging and MRI by enabling longitudinal, whole-brain vascular phenotyping without cranial windows or optical penetration limits.

Together, multiphoton and endoscopic optical imaging provide cellular and regional mechanistic insight, while ultrasound localization microscopy enables global, quantitative assessment of microvascular organization and flow. In murine neuroHIV models such as EcoHIV, these approaches could be integrated with MRI-based structural and perfusion measures to dissect how HIV-associated inflammation and endothelial dysfunction alter neurovascular coupling and cerebral microcirculation across aging. Such multimodal pipelines are particularly important given the strong dependence of hemodynamic signals on behavioral and arousal state, which has been shown to substantially influence vascular and hemodynamic readouts in awake mice (Winder et al., 2017; Turner et al., 2020).

Photoacoustic and optoacoustic imaging represent a particularly compelling modality for studying brain aging because they bridge optical sensitivity to hemoglobin-based metabolism with ultrasound-level penetration and spatial resolution (Park et al., 2025; Mirg et al., 2025).By exploiting the differential absorption spectra of oxy- and deoxyhemoglobin, photoacoustic imaging enables label-free, quantitative mapping of cerebral blood volume, oxygen saturation, and microvascular architecture at depths inaccessible to purely optical approaches (Park et al., 2025). In preclinical systems, transcranial and cranial-window photoacoustic tomography has demonstrated whole-brain and mesoscale vascular imaging through the intact skull, while hybrid photoacoustic–ultrasound platforms allow simultaneous structural, hemodynamic, and functional assessment (Estrada et al., 2025). These capabilities are directly relevant to aging biology, where microvascular rarefaction, impaired oxygen delivery, endothelial dysfunction, and altered neurovascular coupling are central drivers of cognitive decline and neurodegeneration (Santisteban and Iadecola, 2025). Recent advances, including transparent ultrasound transducers, skull-compatible acquisition geometries, and integration with awake-state optical neuroimaging, now permit longitudinal, minimally invasive tracking of hemodynamic and vascular aging trajectories in mice without repeated craniotomy or exogenous contrast (Mirg et al., 2025). Importantly, photoacoustic readouts provide a quantitative complement to BOLD fMRI by directly measuring hemoglobin oxygenation rather than relying on relative signal changes, reducing ambiguity in aging and disease contexts characterized by altered vascular reactivity (Rathbone and Fu, 2024; Mirg et al., 2022).When combined with diffusion and perfusion MRI, multiphoton microscopy, and terminal whole-brain clearing, photoacoustic imaging offers a scalable pathway to link cellular-scale vascular pathology with systems-level brain aging phenotypes, positioning it as a critical component of next-generation longitudinal imaging pipelines in neuroHIV and aging research.

## Future directions: a longitudinal, multiscale imaging agenda for NeuroHIV aging

### Why same-animal longitudinal imaging is essential

NeuroHIV in the antiretroviral therapy era is characterized by subtle, regionally heterogeneous changes that unfold over long timescales, making them difficult to capture with cross-sectional study designs. Between-animal comparisons are often underpowered to detect small effect sizes and can confound infection-related changes with baseline differences in immune tone, vascular biology, and intrinsic brain organization. In contrast, longitudinal imaging enables each animal to serve as its own control, substantially increasing sensitivity to incremental change and reducing inter-individual variance (Ball and Seal, 2019). This approach permits trajectory-based outcomes, such as slopes, inflection points, and variability over time, that align naturally with contemporary frameworks for brain aging and neurocognitive decline.

### EcoHIV as a prioritized platform for whole-brain aging phenotypes

Among available murine neuroHIV models, EcoHIV is particularly well suited for longitudinal imaging studies of brain aging. The model combines virologic relevance, preserved immune competence, and compatibility with high-field MRI and optical imaging approaches, while remaining scalable for lifespan and interventional studies (Gu et al., 2018; Potash et al., 2005; Xie et al., 2024; Kim et al., 2024). Importantly, the primary limitation is not technical feasibility but the absence of standardized, published *in vivo* imaging pipelines that can be replicated across laboratories and systematically linked to behavioral and molecular measures of aging. Addressing this gap would position EcoHIV as a powerful platform for defining imaging-derived brain aging phenotypes in neuroHIV.

### Toward a pragmatic multimodal imaging pipeline

A feasible future workflow would integrate complementary imaging modalities across spatial and temporal scales. Such a pipeline could include baseline structural and diffusion MRI, with or without perfusion imaging, prior to infection or therapeutic manipulation, followed by repeated imaging at prespecified intervals spanning infection chronicity and aging stages (Li et al., 2011; Zhang and Li, 2013). Subsets of animals could undergo targeted optical phenotyping using cranial windows, endoscopic approaches, or multiphoton microscopy to interrogate neurovascular coupling, microglial dynamics, and regional circuit function (Winder et al., 2017; Turner et al., 2020).

Terminal whole-brain clearing with light-sheet microscopy, registered to a three-dimensional atlas framework, would then enable alignment of cellular and vascular phenotypes with *in vivo* imaging signals (Han et al., 2024; Kronman et al., 2024). Integration with standardized behavioral testing and molecular aging measures, including epigenetic, metabolomic, and immunosenescence markers, would allow direct assessment of coupling between systemic aging and brain-specific trajectories.

Critically, the proposed imaging pipeline is designed to generate measures directly translatable to human neuroHIV cohorts. Diffusion MRI metrics (e.g., fractional anisotropy), volumetric changes, and perfusion measures have clear analogues in human studies, while emerging vascular and optical imaging approaches may inform next-generation biomarkers of neurovascular dysfunction. This alignment ensures that animal-derived imaging phenotypes can be integrated with longitudinal human datasets to support biomarker development and therapeutic targeting.

## Conclusion

The field of neuroHIV research has reached an inflection point. In the antiretroviral therapy era, HIV-associated brain injury is best understood as a problem of aging and trajectories rather than acute pathology, yet most animal studies continue to rely on cross-sectional endpoints. Primary *in vivo* imaging studies in SIV-infected macaques demonstrate that longitudinal diffusion and perfusion MRI can quantify within-animal changes across infection stages, providing a critical template for aging-relevant inference. Parallel murine studies, including manganese-enhanced MRI in humanized mouse models, establish that quantitative brain imaging signals can be detected in HIV-relevant small-animal contexts. However, systematic, longitudinal *in vivo* whole-brain neuroimaging remains limited in widely used murine neuroHIV systems, particularly EcoHIV, leaving a substantial gap between mechanistic promise and implementation.

Addressing this gap will require coordinated advances in study design that prioritize repeated, within-animal imaging across complementary spatial scales. In addition to standardized longitudinal MRI and diffusion pipelines, emerging optical approaches are likely to play a central role. Repeated multiphoton imaging enables longitudinal tracking of microglial dynamics, synaptic structure, and neurovascular coupling with cellular precision, while photoacoustic and optical endoscopic methods provide minimally invasive access to deep brain regions and vascular networks that are otherwise inaccessible to conventional optical microscopy. When integrated with awake-state physiology, whole-brain three-dimensional atlasing, and terminal clearing approaches, these modalities offer a practical path to bridge micro- and macro-scale mechanisms over the course of infection and aging.

Establishing reproducible imaging-derived brain aging phenotypes in EcoHIV and related murine models, grounded in repeated multiphoton and endoscopic imaging and anchored by longitudinal MRI, would enable mechanistic tests of inflammation, vascular dysfunction, and viral persistence as drivers of accelerated brain aging, including neuroinflammation, microglial activation, and neurovascular dysfunction. Critically, such phenotypes would create translationally aligned endpoints that can inform longitudinal neuroimaging studies and intervention trials in aging people with HIV.
